# Altering Calcium Sensitivity in Heart Failure: A Crossroads of Disease Etiology and Therapeutic Innovation

**DOI:** 10.3390/ijms242417577

**Published:** 2023-12-17

**Authors:** Nancy S. Saad, Mohammed A. Mashali, Steven J. Repas, Paul M. L. Janssen

**Affiliations:** 1 Department of Physiology and Cell Biology, College of Medicine, The Ohio State University, Columbus, OH 43210, USA; janssen.10@osu.edu; 2 Dorothy M. Davis Heart and Lung Research Institute, The Ohio State University, Columbus, OH 43210, USA; 3 Department of Pharmacology and Toxicology, Faculty of Pharmacy, Helwan University, Cairo 11795, Egypt; 4 Department of Surgery, Faculty of Veterinary Medicine, Damanhour University, Damanhour 22514, Egypt; 5 Department of Emergency Medicine, Wright State University Boonshoft School of Medicine, Dayton, OH 45324, USA; steven.repas@wright.edu

**Keywords:** heart failure, calcium sensitivity, rate constant, troponin C, arrhythmias

## Abstract

Heart failure (HF) presents a significant clinical challenge, with current treatments mainly easing symptoms without stopping disease progression. The targeting of calcium (Ca^2+^) regulation is emerging as a key area for innovative HF treatments that could significantly alter disease outcomes and enhance cardiac function. In this review, we aim to explore the implications of altered Ca^2+^ sensitivity, a key determinant of cardiac muscle force, in HF, including its roles during systole and diastole and its association with different HF types—HF with preserved and reduced ejection fraction (HFpEF and HFrEF, respectively). We further highlight the role of the two rate constants k_on_ (Ca^2+^ binding to Troponin C) and k_off_ (its dissociation) to fully comprehend how changes in Ca^2+^ sensitivity impact heart function. Additionally, we examine how increased Ca^2+^ sensitivity, while boosting systolic function, also presents diastolic risks, potentially leading to arrhythmias and sudden cardiac death. This suggests that strategies aimed at moderating myofilament Ca^2+^ sensitivity could revolutionize anti-arrhythmic approaches, reshaping the HF treatment landscape. In conclusion, we emphasize the need for precision in therapeutic approaches targeting Ca^2+^ sensitivity and call for comprehensive research into the complex interactions between Ca^2+^ regulation, myofilament sensitivity, and their clinical manifestations in HF.

## 1. Introduction

Heart failure (HF) is a serious and often progressive clinical syndrome, classified as one of the major types of cardiovascular disease. HF occurs when the heart is unable to adequately fill with or pump out enough blood to fulfill the body’s requirements. It results from compromised cardiac pump performance that has, as a basis, a reduction in the contractile function and performance of the cardiac myocyte. Around 6.2 million adults aged 20 or older in the United States are estimated to have HF, which accounts for approximately 2.4% of the country’s adult population [1]. The prevalence of HF escalates with age, with the most significant occurrence observed in individuals aged 65 and above. It is noteworthy that the prevalence of HF may be underestimated, as many people with the condition may not be diagnosed or may have asymptomatic or mild forms of the disease [2].

HF can be principally divided into two categories, each with distinct pathophysiological origins. This classification relies on crucial factors, including ejection fraction (EF), natriuretic peptide levels, and the existence of structural heart disease and diastolic dysfunction. The significance of these categorizations rests on their correlations with varying patient demographics, co-existing conditions, and responses to treatment. HF with reduced ejection fraction (HFrEF), also known as systolic HF, is recognized when the EF is 40% or less [3,4]. This condition is characterized by the ventricles’ inability to contract forcefully, which often leads to eccentric hypertrophy of the ventricles, resulting in decreased diastolic volume due to persistent increased resistance or infiltrative disease [5]. Common causes include idiopathic factors, viral infections, alcohol use, chemotherapy, and valvular disease. The majority of current HF treatments are designed for and are most effective in HFrEF. These treatments typically aim to reduce preload, increase contractility, control heart rate, and prevent cardiac remodeling. On the other hand, HF with preserved ejection fraction (HFpEF), also referred to as diastolic HF, arises from the ventricles’ inability to relax, with an EF equal to or greater than 50% [6]. HFpEF is often the result of concentric hypertrophy caused by chronic damage to the myocardium of the ventricles, leading to ineffective contractility [7]. Common causes include hypertension, amyloidosis, idiopathic factors, sarcoidosis, hemochromatosis, and aortic stenosis [8]. Recent research has highlighted the systemic nature of the HFpEF syndrome and the existence of subphenotypes within the heterogeneous HFpEF syndrome, emphasizing the necessity for therapies that are better targeted towards specific HFpEF subtypes [9].

Another category is HF with mid-range ejection fraction (HFmrEF), sometimes referred to as HFpEF-borderline or HFpEF-improved when the EF in HFrEF rises above 40%. This is recognized when the EF is between 41–49% according to European guidelines [10] or between 40–49% per US guidelines [11]. This category was introduced by the 2016 European Society of Cardiology (ESC) guidelines for HF diagnosis and management and was initially regarded as a grey area between HFpEF and HFrEF.

The complete cure for HF remains a challenging quest for the medical community. Existing treatments largely focus on symptom alleviation, cardiac remodeling reduction, and cardiac function optimization. These strategies, while integral for patient comfort, may fall short in halting HF progression. In response to this unmet need, researchers worldwide are intensifying their efforts to devise innovative therapeutic solutions for HF, a path that requires a thorough understanding of the disease’s fundamental pathophysiology.

Investigations into these mechanisms have spotlighted the critical role of calcium (Ca^2+^) regulation, given its fundamental role in mediating cardiac muscle contractions [12]. Disruptions in Ca^2+^ homeostasis within heart muscle cells, or cardiomyocytes, have been linked to the two primary causes of mortality in HF patients: deteriorating cardiac pump function and arrhythmia onset [13]. These disruptions originate from pathological alterations in the expression and activity of a wide range of Ca^2+^ homeostatic and structural proteins, ion channels, and enzymes.

**Figure 1:**
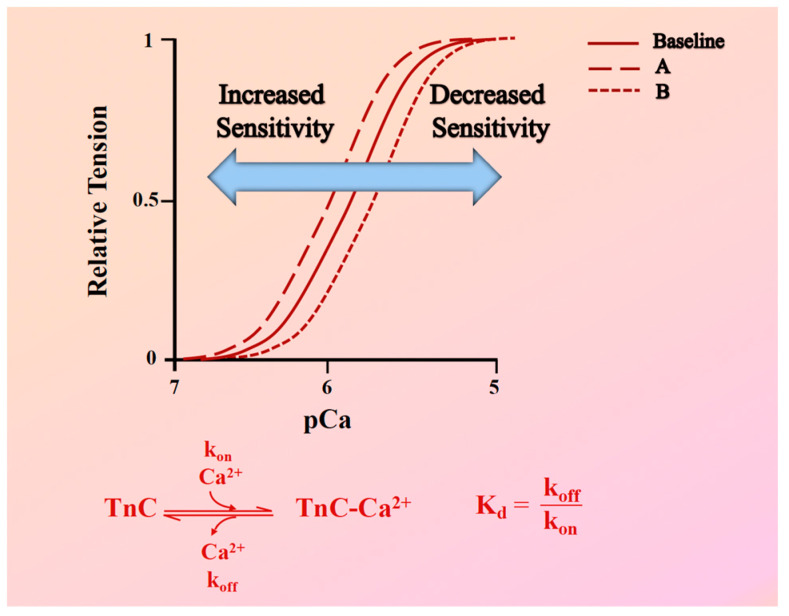
**Assessment of myofilament Ca^2+^ response variability. Top:** Force-pCa^2+^ relationship curves showing variations in myofilament Ca^2+^ sensitivity. The solid line represents the baseline force-Ca^2+^ relationship. Curve (A) demonstrates increased Ca^2+^ sensitivity, indicated by a leftward shift, where a given steady-state force is achieved at lower Ca^2+^ concentrations. Curve (B) demonstrates decreased Ca^2+^ sensitivity, indicated by a rightward shift, requiring higher Ca^2+^ concentrations to generate the same steady-state force. **Bottom**: This panel shows a biochemical representation of the Ca^2+^ binding dynamics to Troponin C (TnC). It includes the calcium association rate (k_on_) and dissociation rate (k_off_) from TnC, alongside a formula illustrating the relationship between the equilibrium dissociation constant (Kd), k_on_, and k_off_.

Central to these irregularities is the alteration in myofilament Ca^2+^ sensitivity. This term, often referred to as Ca^2+^ sensitivity, refers to the variation in force generation at a specified calcium concentration [Ca^2+^], which plays an essential role in determining the contractility of striated muscles. It is essential to emphasize that myofilament Ca^2+^ sensitivity is a key indicator of cardiac muscle performance, particularly when considering pathological conditions such as hypertrophic cardiomyopathy (HCM) and dilated cardiomyopathy (DCM). This myofilament Ca^2+^ sensitivity provides valuable information about the muscle’s ability to generate mechanical force at steady-state. It is typically gauged through the construction of a force-pCa curve, where shifts in this curve to the left or right indicate increased or decreased Ca^2+^ sensitivity, respectively (Figure 1). 

While myofilament Ca^2+^ sensitivity can be indicative of altered dynamic behavior, as a stand-alone assessment, it does not provide a complete picture. A crucial factor in understanding the dynamic behavior of a change in myofilament Ca^2+^ sensitivity is the fact that the equilibrium dissociation constant (Kd) of Troponin C (TnC) is impacted by two parameters. These two parameters are the Ca^2+^ association and dissociation rate constants to and from TnC, respectively (known as k_on_ and k_off_). Both these parameters affect the binding of Ca^2+^ to TnC, which in turn affects muscle contraction and relaxation (Figure 1). As previously discussed by Chung et al. [14], the relationship between k_on_ and k_off_ is vital: while increasing k_on_ can enhance TnC’s Ca^2+^ sensitivity, the actual dynamics of muscle contraction are more complex and are influenced by multiple variables. Factors such as muscle length, frequency of contraction, β-adrenergic stimulation, and membrane permeabilization all impact myofilament Ca^2+^ sensitivity and, consequently, the dynamic contraction of the myocardium. To further dissect these influences, a mathematical model was employed to examine the impact of various parameters, specifically focusing on k_on_ and k_off_ for Ca^2+^ binding to TnC. The model revealed that alterations in these rates can have different outcomes on dynamic twitch kinetics. For instance, increased Ca^2+^ sensitivity via an increased k_on_ would lead to enhanced tension development without necessarily affecting contraction speed, while an identical increase in Ca^2+^ sensitivity via a decreased k_off_ would cause relaxation to slow down but contraction to only be minimally impacted. In addition, if both k_on_ and k_off_ are adjusted simultaneously in the same direction, it could result in no apparent shift in steady-state Ca^2+^ sensitivity but have profound effects on contraction dynamic twitch kinetics of the cardiac muscle [14].

Exploring the genetic terrain, mutations in myofilament proteins play a pivotal role in influencing Ca^2+^ sensitivity, directly affecting the dynamics of muscle contraction and relaxation. While numerous genetic mutations in these proteins have been documented, in-depth studies of their implications on muscle behavior remain limited to only a subset. To fully comprehend the impact of a mutation, it is not sufficient to merely understand its effect on Ca^2+^ sensitivity. Direct observations on the rates of k_on_ and k_off_, as well as twitch force kinetics in intact muscles, are equally essential [14]. Take, for instance, the Troponin I (TnI) R145G HCM mutation. While it is known to elevate Ca^2+^ sensitivity, this effect might primarily be attributed to a decreased k_off_ rate for Ca^2+^ from TnC. Moreover, this mutation has also been shown to alter force and relaxation kinetics [15]. Similarly, mutations in Troponin T (TnT), which is integral to the troponin complex, result in notable shifts in Ca^2+^ sensitivity and muscle dynamics. Some studies suggest these changes could be tied to increased k_off_ rates [16,17]. The β-myosin heavy chain gene, *MYH7*, which is associated with a multitude of mutations in familial HCM patients, presents another layer of complexity. While some mutations in *MYH7* are characterized by alterations in Ca^2+^ sensitivity [18,19], others distinctly influence the muscle’s relaxation kinetics [20,21]. Adding to the intricacy is the E22K mutation in myosin light chain-2 (MLC-2). Found in HCM patients, this mutation yields inconsistent findings. Some studies pinpoint an increased Ca^2+^ sensitivity [22], whereas others discern no noticeable change [23]. To fully recognize cardiac muscle behavior and potential malfunctions in disease states, a multifaceted approach, which considers both steady-state measurements and dynamic factors, particularly the k_on_ and k_off_ rates, is essential.

This review aims to further explore the modifications in Ca^2+^ sensitivity observed in HF and how these changes correlate with the rate of cardiac relaxation and the incidence of diastolic dysfunction. In addition, we aim to compare the possible differences in these alterations between HFpEF and HFrEF. Furthermore, this review will encapsulate an examination of past studies that have concentrated on proteins responsible for Ca^2+^ handling and how the modulation of these proteins could influence Ca^2+^ sensitivity in failing hearts. Importantly, we will shed light on the promising therapeutic targets that have been identified through these studies.

## 2. Overview of Ca^2+^ Cycling and Homeostasis

**Figure 2:**
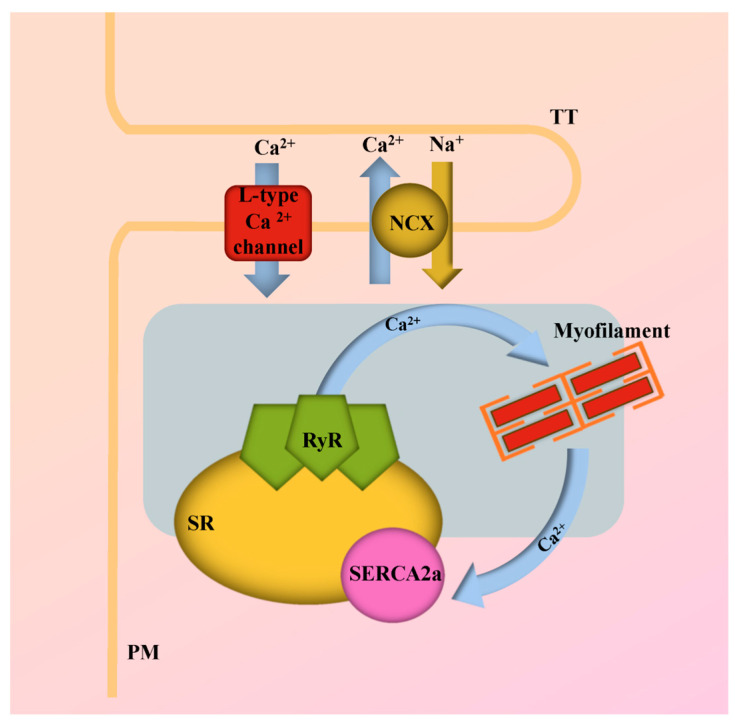
**Calcium-handling in excitation–contraction (EC) coupling**. Schematic overview of key Ca^2+^-handling proteins and their roles in the process of EC coupling. NCX, Na^+^/Ca^2+^ exchanger; PM, Plasma membrane; RyR, Ryanodine receptor; SERCA2a, Sarco/endoplasmic reticulum ATPase type-2a; SR, Sarcoplasmic reticulum; TT, Transverse tubule.

**Figure 3:**
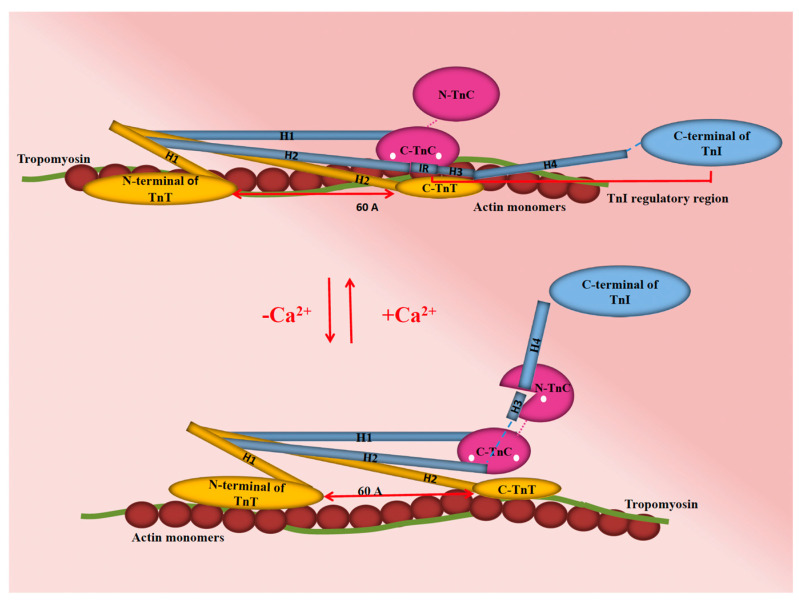
A schematic representation of cardiac troponin’s interaction with the thin filament under two conditions: absence and presence of Ca^2+^ ions (represented as white dots), emphasizing its role in modulating cardiac muscle contraction. In the thin filament OFF state (up), myosin binding sites on actin are obstructed by tropomyosin (green), preventing contraction. Upon Ca^2+^ binding to the C-terminal lobe of troponin C (C-TnC; pink), a key conformational change occurs. The switch peptide of troponin I (TnI), which includes helix H3 (H3; blue), interacts with the regulatory N-terminal lobe of TnC (N-TnC; pink). This interaction triggers the removal of the C-terminal lobe of TnI, allowing tropomyosin to shift azimuthally around the thin filament. Consequently, this shift exposes the myosin binding sites on actin, facilitating muscle contraction. The C-lobe of TnC (C-TnC; pink), which is constantly bound to divalent cations alongside the anchoring region of TnI (blue), plays a vital role in the stability and function of the thin filament complex. TnC, Troponin C; TnI, Troponin I; TnT, Troponin T.

Myocardial contractility is fundamentally governed by the cyclical movement of Ca^2+^ in and out of the cytoplasm of cardiac myocytes. This movement is intricately linked with the Ca^2+^ sensitivity of various proteins present in these cardiac myocytes. As such, Ca^2+^ serves as a key regulator of excitation–contraction (EC) coupling, a process integral to the modulation of systolic and diastolic function in the heart, as illustrated in (Figure 2). The unfolding of EC coupling and its corresponding Ca^2+^ signal transduction is a sequence of four main steps [24]. The first step is initiated by membrane depolarization, which prompts the generation of a Ca^2+^ current, referred to as ICa. This current is the product of L-type Ca^2+^ channels situated in the transverse tubules (T-tubules) of the cardiac myocytes. In the subsequent second step, the Ca^2+^ ions navigate through a narrow junctional area, thereby activating ryanodine receptors (RyR) and giving rise to Ca^2+^ sparks. These sparks represent localized, spontaneous calcium release events within cardiac myocytes. While these sparks significantly augment the original trigger Ca^2+^ signal through a mechanism known as Ca^2+^-induced Ca^2+^ release (CICR), they are distinct from the massive global increase in intracellular calcium that characterizes EC coupling. They are, instead, integral components that contribute to the broader calcium dynamics within the myocyte. In the third step, Ca^2+^ released from the sarcoplasmic reticulum (SR) spreads throughout the cytoplasm. This dispersal of Ca^2+^ binds to TnC, allowing actin–myosin cross-bridging and the thick and thin filaments of the sarcomere to slide past each other, shortening the sarcomere and causing cardiac muscle contraction (Figure 3). Lastly, in the fourth step, for the muscle to relax, Ca^2+^ must come off TnC to cease activation and allow dissociation of thin and thick filaments to occur and relax the muscle. Ca^2+^ ions are recycled back into the SR via Sarco/Endoplasmic Ca^2+^-ATPases (SERCA) or extruded out of the cell mainly via Na^+^/Ca^2+^ exchanger (NCX), with a minor role of the continually active Ca^2+^ channels.

## 3. Alterations in Ca^2+^ Sensitivity in HF

The sensitivity of myofilaments to Ca^2+^ is dynamically influenced by several processes that connect Ca^2+^ cycling to the production of myofilament force. These include Ca^2+^ binding to TnC, the thin filament’s removal of actin–myosin interaction inhibition, and the properties of actin–myosin cross-bridges [25]. Notably, changes in Ca^2+^ sensitivity can occur within each cardiac cycle along with the sarcomere length [26,27]. This change partly drives the immediate adaptation in cardiac output during beat-to-beat alterations in the ventricular filling, the Frank–Starling response [28]. 

Long-lasting regulation of myofilament Ca^2+^ sensitivity is often achieved through the process of phosphorylation [29]. A significant instance of this regulation is the phosphorylation of TnI [30]. The phosphorylation of two N-terminal serines by the cyclic Adenosine Monophosphate (cAMP)-dependent protein kinase A (PKA) reduces myofilament Ca^2+^ sensitivity and contributes to the beta agonists’ positive lusitropic effect [31,32]. These same serines are also phosphorylated by protein kinase D (PKD) [33], enabling multiple signaling pathways to regulate force production’s Ca^2+^ dependence through this mechanism. Altered Ca^2+^ sensitivity then acts as the primary stimulus for impaired contractility in cardiomyocytes [34].

At the end-stage of HF, various cardiac contractile proteins often undergo changes in their isoform composition and phosphorylation status [35]. Modifications have been noted in the isoform composition of proteins, such as TnT [36,37,38,39] and myosin [40,41], as well as the degradation of proteins, including MLC-2, TnT, and TnI [42]. Cardiac overload can trigger changes in the hormone-mediated activation of PKA and Protein Kinase C (PKC) via agents like noradrenalin, endothelin, or angiotensin [43,44,45,46]. These changes could potentially alter the phosphorylation status of contractile proteins. Furthermore, an increase in PKC [47] and protein phosphatase [48] activities has been detected in failing human hearts, which could result in the phosphorylation and dephosphorylation of contractile proteins, respectively. 

Ca^2+^ sensitivity has been reported to either increase or decrease in failing myocardium depending on the etiology of the disease [49]. There is now a broad agreement that HCM mutations typically increase the Ca^2+^ sensitivity of ATPase activation [50,51]. By contrast, during episodes of acute myocardial ischemia, myofilament Ca^2+^ sensitivity decreases significantly, predominantly due to the combined effects of acidic pH and elevated phosphate levels (consequent to the decline in high-energy phosphates) [52,53,54]. Even after the restoration of the intracellular environment, myofilament Ca^2+^ sensitivity remains reduced in post-ischemic or “stunned” myocardium. This persistent decrease likely results from modifications to contractile proteins or proteolytic damage [55,56]. 

A considerable number of studies have illuminated the fact that the sensitivity of the contractile apparatus to Ca^2+^ is heightened during the end-stages of human HF, highlighting it as a notable risk factor for ventricular tachyarrhythmias development, a common occurrence in this condition [39,40,57,58,59]. Alternative explanations have been given for the origin of the increased Ca^2+^ responsiveness in human HF. According to Morano et al. [40], the increased Ca^2+^ sensitivity of the contractile apparatus is due to the expression of atrial light chain 1 (ALC-1) in the left ventricle. Margossian et al.’s [60] investigation provides evidence that proteolytic breakdown of MLC-2 may be an important mechanism that contributes to contractile failure in idiopathic dilated cardiomyopathy (IDC). Myofilaments that have lost MLC-2 should be more sensitive to Ca^2+^ and, while producing the same maximum force, have elevated submaximal force. They should have a reduced actomyosin ATPase rate and a reduced maximum unloaded shortening velocity. 

Most studies on the contractile apparatus have focused on troponin, the Ca^2+^-dependent regulator of myofibrillar activity. In particular, it has been noted that phosphorylation of TnI as a result of β-adrenergic/PKA activity effectively desensitizes myofilaments to Ca^2+^, thereby playing a pivotal role in improving both systolic and diastolic performance [61,62]. Changes in cardiac TnI phosphorylation status have been reported in failing human hearts [63,64,65] and may reflect changes in the balance between kinase and phosphatase activities. The examination of muscle samples from failing human hearts has revealed notably low levels of cardiac TnI phosphorylation alongside high Ca^2+^ sensitivity [66,67,68,69]. Wolff et al. [67] reported an increase in myofibrillar Ca^2+^ sensitivity of isometric tension in a canine model of DCM produced by chronic rapid pacing, likely due at least in part to chronic reductions in β-adrenergic-mediated (PKA-dependent) phosphorylation of myofilament regulatory proteins. This hypothesis is further supported by the fact that both β-adrenergic receptor density and adenylate cyclase activity are commonly downregulated in HF [70,71,72,73]. Such downregulation could result in decreased PKA-dependent phosphorylation of myofilament regulatory proteins, which may be the mechanism behind the observed increase in Ca^2+^ sensitivity of isometric tension.

In addition, re-expression of a fetal TnT isoform was observed in end-stage failing myocardial tissue exhibiting increased Ca^2+^ responsiveness of the contractile apparatus [39]. Additionally, a decrease in the phosphorylation level of TnC has been detected in HF cases [74], providing yet another potential explanation for the observed reduction in contractile function in failing hearts [75]. Some but not all of the changes in myofilament activity and regulation predicted from these studies have been seen in myofilament preparations from human cardiomyopathy [76,77]. 

Since these previous studies concentrated on a single factor, the question remains whether the increased Ca^2+^ sensitivity of the contractile apparatus is attributed to one of the above-mentioned protein changes or is the complex resultant of several combined protein changes. Table 1 provides a summary of previous studies conducted to identify the factors influencing changes in Ca^2+^ sensitivity during cardiac diseases.

The concept of enhanced Ca^2+^ sensitivity implies a more reactive state of contractile proteins to lower Ca^2+^ concentrations, a condition that holds both potential benefits and drawbacks. Viewed from one angle, it might be beneficial during systole because it allows the heart to generate more robust contractions even in the face of lower Ca^2+^ levels. This could potentially offset other irregularities present in a failing heart. Conversely, this heightened Ca^2+^ sensitivity can be problematic during diastole. For the heart to effectively relax and refill with blood, it necessitates the rapid removal of Ca^2+^ from muscle cells. However, if the myofilaments are overly sensitive to Ca^2+^, they may not relax adequately even as Ca^2+^ levels drop, limiting the heart’s ability to refill with blood. This impaired refilling process is known as diastolic dysfunction, which is a common feature of HF. Thus, while an increased sensitivity to Ca^2+^ could partially offset anomalies in the systolic Ca^2+^ transient of a failing heart, it might concurrently harm diastolic function. Again, it is critical to know whether the enhanced Ca^2+^ sensitivity results from a predominantly increase in k_on_ or a decrease in k_off_, but this critical information is often not investigated, leaving much room for uncertainty in extrapolating findings on steady-state Ca^2+^ sensitivity to altered dynamic behavior during in vivo contractions.

Another crucial consideration is the propensity for increased Ca^2+^ sensitivity to be a trigger for hazardous arrhythmias, leading to sudden cardiac death. Statistics reveal that sudden cardiac death accounts for 30–50% of fatalities among HF patients, and most of these deaths are associated with ventricular tachycardia [82]. This condition can be brought on by spontaneous electrical activity within the cardiomyocytes. In certain scenarios, a spontaneous action potential may be initiated by a phase of depolarization that occurs during the downstroke of the action potential, a phenomenon termed early afterdepolarization (EAD). Though the exact mechanisms leading to the generation of EADs continue to be a topic of debate and may differ across various settings, it is generally agreed upon that many EADs originate from the improper re-opening of L-type Ca^2+^ channels (LTCCs) or other depolarizing currents [83]. Abnormal Ca^2+^ homeostasis promotes arrhythmogenesis via delayed afterdepolarizations (DADs). These events can be triggered, at least partially, due to increased myofilament Ca^2+^ sensitivity [84]. This array of findings collectively supports a thought-provoking hypothesis: strategies designed to reduce myofilament Ca^2+^ sensitivity could provide a novel anti-arrhythmic approach. If proven effective, these strategies could potentially transform the existing treatment paradigm for HF.

Alterations in Ca^2+^ sensitivity and disrupted Ca^2+^ homeostasis could also likely induce various cellular processes, leading to morphological changes in the heart [85]. Earlier findings indicate that an elevated Ca^2+^ sensitivity in end-stage failing myocardium could cause the muscle to be hypercontractile, thereby increasing ATP consumption [86]. Over time, this increased energy demand can lead to various adaptive and maladaptive responses in the heart. Some potential impacts on cardiac morphology may manifest as hypertrophy, fibrosis, cellular changes, chamber remodeling, mitochondrial dysfunction, and apoptosis. Moreover, unique combination of properties in HCM TnT mutants including reduced maximal activation, depressed cooperativity, and, at an equimolar ratio with wild type troponin, diminished Ca^2+^ sensitivity may lead to a dilated heart condition rather than the expected hypertrophic condition [87]. Interestingly, HCM TnT mutants exhibit a unique combination of traits: reduced maximal activation, decreased cooperativity, and diminished Ca^2+^ sensitivity when present in an equimolar ratio with the wild-type troponin. Contrary to expectations, these properties might steer the heart towards a dilated phenotype instead of the typical hypertrophic condition.

## 4. Ca^2+^ Sensitivity Changes in HFpEF and HFrEF

About half of all HF patients are diagnosed with HFpEF, and the other half with HFrEF [88]. HFrEF often emerges from primary myocardial injuries such as myocardial infarction, viral cardiomyopathy, genetic anomalies, or cardiotoxicity. In contrast, HFpEF typically arises from external insults, often related to other health conditions, such as aortic stenosis and hypertension, which subsequently result in myocardial dysfunction [89]. Exploring the molecular details reveals that the two HF phenotypes exhibit unique patterns, with cardiac remodeling being a common feature characterized by changes in the phosphorylation of myofilament proteins, particularly regulatory proteins [75,90,91]. While there is a depth of understanding regarding these changes in HFrEF, insights into HFpEF remain relatively limited [75,90,91]. For instance, the protein TnI, pivotal for myofilament Ca^2+^ sensitivity, has been subject to varying results across studies. Some indicate hyperphosphorylation in HFrEF, while others suggest hypophosphorylation. Similar inconsistencies are reported for other regulatory proteins [92,93,94,95,96,97], and the exact reasons for such divergences are yet to be identified.

Central to HF’s pathology is the alteration in Ca^2+^ handling, often stemming from myofilament regulatory protein phosphorylation. For instance, in the case of HFrEF, cardiac dysfunction is intrinsic to the cardiomyocytes, largely due to abnormalities in Ca^2+^ handling and disturbances in EC coupling. This includes a reduced systolic Ca^2+^ transient amplitude with a slower rate-of-rise, correlating with decreased cardiomyocyte shortening and delayed relaxation onset. A slower decay of the Ca^2+^ transient exacerbates this by impairing relaxation kinetics during diastole. At a molecular level, these phenomena are attributed to complex changes in the expression, localization, and function of key Ca^2+^ handling proteins, particularly SERCA, Phospholamban (PLN), NCX, and RyR [98]. The decrease in SERCA expression or PLN phosphorylation, often observed in HFrEF, hinders SR Ca^2+^ reuptake and delays relaxation, thereby reducing SR Ca^2+^ content [99]. This is compounded by an increased expression of NCX, which competes with SERCA for Ca^2+^, and heightened RyR and inositol 1,4,5-trisphosphate receptor type 2 (IP3R2) channel activity, leading to diastolic SR Ca^2+^ leak [100]. These alterations are further exacerbated by structural changes in the t-tubule and SR network due to elevated wall stress [101], leading to a decoupling of the calcium voltage-gated channel 1.2 (CaV1.2) from RyR [102] and the emergence of ‘orphaned’ RyR channels as foci for diastolic Ca^2+^ release events [103].

In contrast, the alterations in Ca^2+^ handling associated with HFpEF are less well-defined, partly due to the limited availability of cardiac tissue from HFpEF patients and the lack of comprehensive animal models. However, in HFpEF-related models, cardiomyocyte Ca^2+^ transients are often found to be normal or even enhanced [104,105,106,107,108,109,110], suggesting an adaptive phase where Ca^2+^ flux shifts towards cardiomyocyte Ca^2+^ accumulation [111]. This adaptation may involve excessive Ca^2+^ entry through CaV1.2 and transient receptor potential (TRP) channels [112,113,114], along with increased SR Ca^2+^ release through RyR [104,105,110] and IP3R2 [109], enhancing Ca^2+^ cycling and contraction. Yet, without a simultaneous enhancement of SERCA activity, this leads to elevated diastolic Ca^2+^, preserved or enhanced Ca^2+^ transient amplitude, but slower Ca^2+^ reuptake kinetics and impaired relaxation. These changes become more pronounced under elevated stimulation frequencies, contributing to the chronotropic intolerance and reduced exercise capacity characteristic of HFpEF patients. This observation is critical, given that exercise intolerance is a prominent feature of HFpEF, often associated with impaired cardiac responses to β-adrenergic stimulation. Unlike HFrEF, where β-blockers have shown clinical benefits, their efficacy in HFpEF patients is less clear. This difference might be due to the distinct features of Ca^2+^ regulation in response to β-adrenergic stimulation in HFpEF, where components of the Ca^2+^ handling apparatus do not exhibit the same level of dysregulation as in HFrEF. Notably, both HF phenotypes might experience impairments in Ca^2+^ removal pathways [99]. Therefore, therapeutically targeting cardiomyocyte Ca^2+^ homeostasis emerges as a promising avenue to enhance both systolic and diastolic functions in HF patients.

Differences in Ca^2+^ handling between HFpEF and HFrEF have been observed, but deeper exploration into the variations in Ca^2+^ sensitivity remains somewhat underexplored. The sympathetic nervous system plays a pivotal role in modulating Ca^2+^ sensitivity in both HFpEF and HFrEF, although its mechanisms and impacts vary between the two. In HFpEF, sympathetic overactivity, often linked to comorbid conditions like hypertension, leads to augmented β-adrenergic signaling [115]. This signaling increases cAMP levels, leading to enhanced PKA activity. PKA phosphorylates key myofilament proteins, such as TnI and cardiac myosin binding protein C (cMyBP-C), thus increasing Ca^2+^ sensitivity [70,71,72,73]. This hyperphosphorylation may contribute to the increased myofilament Ca^2+^ sensitivity observed in HFpEF [116], aligning with the heightened ventricular stiffness and impaired relaxation characteristic of this condition. Conversely, in HFrEF, the sympathetic nervous system’s influence is more complex. Chronic sympathetic activation, often resulting from reduced cardiac output, leads to sustained β-adrenergic stimulation [117]. However, prolonged exposure to high catecholamine levels can lead to β-adrenergic receptor desensitization and downregulation, causing a blunted response to sympathetic stimulation [118]. This can result in altered Ca^2+^ handling and reduced Ca^2+^ sensitivity due to changes in the phosphorylation state of myofilament proteins. The interplay of these mechanisms underlines the sympathetic nervous system’s differential impact on Ca^2+^ sensitivity and cardiac function in HFpEF and HFrEF, offering insights into potential therapeutic targets for modulating Ca^2+^ handling in these distinct heart failure phenotypes.

In research by Hegemann et al. [116], strong links emerged between right ventricle (RV) remodeling in HFpEF and notable shifts in RV cardiomyocyte Ca^2+^ balance, particularly a rise in myofilament Ca^2+^ sensitivity. This shift may be attributed to the hyperphosphorylation of cMyBP-C. Upon activation via PKA phosphorylation, cMyBP-C has an increased propensity to bind to actin over myosin S2. Such a modification is pivotal in enhancing the Ca^2+^ sensitivity of the thin filament [119,120]. Further adding to this, a study on an experimentally created HFpEF model that closely reflected patient conditions showed marked changes in the titin protein, leading to its rigidity. This rigidity was attributed to both a shift in titin’s isoform composition and its phosphorylation patterns. These alterations are believed to account for the heightened stiffness detected in the left ventricle (LV) in this model. Complementing these findings was evidence pointing to myofilament proteins being less phosphorylated, coupled with an increased Ca^2+^ sensitivity. This hints at malfunctions at the sarcomere level as potential early indicators in HFpEF onset [121].

Parallel to these discoveries, research led by Røe et al. [122] dissected the complexities of diastolic dysfunction seen in concentric hypertrophy, a defining characteristic of HFpEF. This study highlighted that such dysfunction is not shaped by a single factor. While passive myocardial stiffening has a part to play, the spotlight was also cast on positive shifts in Ca^2+^ cycling. This emphasized the improvement in diastolic Ca^2+^ management and the unchanged Ca^2+^ sensitivity. In a separate investigation [123] that probed the cardiac gene expression patterns in patients with HFrEF and HFpEF using biopsy samples, distinguishing molecular profiles came to the forefront. Both sets of patients exhibited unique molecular imprints, particularly in genes tied to the somatotropic axis, Ca^2+^ management, and adrenergic signaling. Notably, HFrEF patients displayed a significant reduction in cardiac SERCA2 levels relative to their HFpEF counterparts. This suggests that Ca^2+^ transients could potentially be quicker in HFpEF, leading to enhanced Ca^2+^ reuptake and unchanged intracellular Ca^2+^ sensitivity [123].

At the level of the sarcomere, there are compelling data suggesting that the actin–myosin filaments play a role in HFpEF. The relaxation of these filaments is governed by diastolic [Ca^2+^]i levels and their responsiveness to Ca^2+^. Elevated sensitivity to Ca^2+^ in the myofilament, often a result of cardiac TnI’s hypophosphorylation, has been observed in cases of HFpEF [121]. Additionally, this heightened Ca^2+^ sensitivity in the myofilament has been linked to the diastolic dysfunction seen in hypertrophic cardiomyopathy, a condition often triggered by mutations in sarcomeric genes [124,125]. It is also noteworthy that the increased resting tension detected in HFpEF myocytes is associated with diminished levels of protein kinase G (PKG). This reduction may hinder relaxation by decreasing the phosphorylation of molecules like titin, cardiac TnI, and PLN [126,127]. The idea that defective calcium/calmodulin-dependent protein kinase II (CaMKII) phosphorylation of titin might be involved has also been put forward [128]. Thus, overall, while there are several factors that can influence diastolic function, it is vital to acknowledge the potential impact of irregularities in Ca^2+^ signaling and sensitivity.

## 5. Manipulating Ca^2+^ Sensitivity for Therapeutic Gain

The current market’s predominant drug class for enhancing cardiac muscle contractility (positive inotropes) predominantly functions via β-adrenergic pathways, including catecholamines and phosphodiesterase inhibitors, such as dobutamine, milrinone, and inamrinone [129]. On a molecular level, these substances mainly augment inotropy by elevating systolic Ca^2+^ levels. However, the leading theories behind the ineffectiveness of existing inotropic treatments highlight numerous drawbacks: they amplify activator Ca^2+^, exacerbate arrhythmias, trigger maladaptive Ca^2+^-dependent signaling cascades, and heighten myocardial oxygen consumption, resulting in reduced cardiac efficiency [130].

On the other hand, augmenting the Ca^2+^ sensitivity of the contractile machinery without altering systolic Ca^2+^ levels is a promising alternative, as initially proposed by Solaro et al. [131] and subsequently demonstrated through viral gene delivery in myocardial infarcted mice [132]. This approach employs “Ca^2+^ sensitizers”, a class of molecules gaining clinical attention for over two decades [131,132,133,134,135]. The mechanisms these substances use vary widely and range from direct motor protein activators like myosin, enhancers of cross-bridge-generated force, to agents amplifying Ca^2+^– TnC binding and its subsequent effects. Many of these drugs also exhibit additional effects, such as inhibiting cAMP phosphodiesterase 3A (PDE3A), contributing to their vasodilation/venodilation properties, and Ca^2+^-dependent increases in heart rate and contractility. Despite the theoretical promise and successful animal model applications of Ca^2+^ sensitization, there is a notable lack of FDA-approved pharmaceuticals focusing on modulating Ca^2+^ sensitivity for chronic HF treatment [136]. Currently, three compounds—bepridil, levosimendan, and pimobendan—are prescribed for HF outside the United States. Levosimendan failed to gain FDA approval, and the use of bepridil was discontinued due to these compounds’ tendency to cause fatal cardiac arrhythmias, particularly torsade de pointes.

Understanding these mechanisms provides valuable insights into the disease’s etiology. Elevated myofilament Ca^2+^ sensitivity has been identified in numerous studies on end-stage HF patients [58,65,137], marking it as a significant risk factor for ventricular tachyarrhythmias, a frequent HF complication [84]. These findings together build a strong case for the hypothesis that reducing myofilament Ca^2+^ sensitivity might offer a transformative anti-arrhythmic strategy, revolutionizing the treatment landscape for HF. Despite this, Ca^2+^ de-sensitizing agents are currently rare, with their potential only recently coming to light. Blebbistatin (BLEB), which acts as an actin–myosin uncoupler, has shown its capability to adjust the dependency of force development on Ca^2+^ to the right, with negligible impacts on cardiac ion channels [138,139]. Baudenbacher et al. [84] replicated this phenomenon, illustrating that BLEB lowers myofilament Ca^2+^ sensitivity in TnT mutant mice and counteracts the Ca^2+^ sensitizing impact of EMD. In line with these findings, BLEB effectively halted the heightened incidence of ventricular tachycardia across all groups with increased Ca^2+^ sensitivity (TnT mutants and those treated with EMD). This marks the first instance demonstrating that decreasing Ca^2+^ sensitivity in myofilaments holds anti-arrhythmic properties, potentially offering a therapeutic advantage for individuals suffering from hypertrophic cardiomyopathy.

Expanding upon this perspective, the introduction of cardiac myosin inhibitors such as mavacamten represents a significant breakthrough in targeting the fundamental mechanisms of HCM. Mavacamten, a pioneering, targeted, and cardiac-specific myosin inhibitor, has gained approval from the US Food and Drug Administration for the treatment of adults with HCM [140]. This marks a crucial shift in the therapeutic approach, focusing on the underlying pathophysiological processes of the condition. Clinical trials have highlighted the efficacy of mavacamten, demonstrating its capacity to improve cardiac output and exercise capability, alongside a notable reduction in LV outflow tract (LVOT) gradients in HCM patients [141,142]. The drug’s mechanism of action, which involves normalizing the balance of “on” and “off” myosin heads within cardiac muscle cells, directly addresses the hypercontractile nature of HCM [141,143]. This modulation, by alleviating LVOT obstruction and reducing LV filling pressures, effectively addresses two of the primary challenges encountered in the management of HCM.

Crucially, mavacamten’s ability to reduce Ca^2+^ sensitivity emerges as a pivotal aspect of its therapeutic profile [143]. This reduction in Ca^2+^ sensitivity, coupled with its impact on alleviating diastolic dysfunction, could position mavacamten as a promising agent in the broader context of HF treatment. It offers a novel approach, especially in HF subtypes where abnormal Ca^2+^ sensitivity and diastolic dysfunction are prevalent and contribute significantly to disease progression. By targeting these core pathophysiological elements, mavacamten extends beyond just providing symptomatic relief, suggesting a potential for improved patient outcomes in various HF scenarios. This shift towards modulating fundamental cardiac mechanics with drugs like mavacamten highlights the need for ongoing research and clinical trials. It is imperative to investigate and comprehend the full spectrum of benefits offered by cardiac myosin inhibitors further. Such research is crucial in broadening our understanding of their potential applications across diverse HF pathologies. The continued exploration of mavacamten and similar therapies holds the promise of revolutionizing HF management, offering more effective, targeted treatments that could significantly alter the course of the disease and improve patients’ quality of life.

## 6. Conclusions

The alterations in Ca^2+^ sensitivity and their relationship with the incidence of cardiac relaxation rate and diastolic dysfunction in HF present significant clinical implications. A deeper understanding of these changes can guide the development of innovative therapeutic strategies targeting Ca^2+^ handling and sensitivity. Nonetheless, further research is warranted to fully elucidate the complex interplay between Ca^2+^ regulation, myofilament sensitivity, and their implications in different forms of HF. This knowledge can pave the way for more effective and targeted treatments for this debilitating disease.

## Figures and Tables

**Table 1:** Various pathways and targets contribute to altered Ca^2+^ sensitivity during cardiac disorders.

Cardiac Disease	Model	Pathway/Target	Variations in Ca^2+^ Sensitivity	Reference
Ischemic and DCM	Human-skinned LV papillary muscle fibers	Expression of ALC-1 in the LV in addition to the essential VLC-1	Increased	[40]
IDC	Human right and left ventricular tissues	Proteolytic break down of MLC-2	Increased	[60]
DCM	Human left ventricular myocytes	Reduction of the β-adrenergically mediated phosphorylation of TnI via PKA	Increased	[58]
HF	Human mechanically isolated Triton-skinned single myocytes from LV	MLC-2 phosphorylation was significantly lower	Increased	[57]
End-stage HF	Human left ventricular myocytes	Increased percentage of dephosphorylated MLC-2 and TnI	Increased	[65]
Mitral or aortic valvular disease	Human left ventricular and atrial skinned myocytes	Re-expression of a fetal TnT	Increased	[39]
FHC	Human genetic screening	AMPK γ_2_ mutations	Increased	[78]
DCM	Canine left ventricular myocytes	Chronic reductions in β-adrenergic-mediated (PKA-dependent) phosphorylation of myofilament regulatory proteins such as TnI and/or C-protein.	Increased	[67]
HF	Rat cardiomyocytes	Low levels of TnI phosphorylation	Increased	[68]
MI	Pig left ventricular myocytes	Reduced TnI phosphorylation	Increased	[66]
HCM	Porcine left ventricular papillary muscle strips	Mutations in TnC	Increased	[79]
Ischemic and IDC	Human left ventricular skinned-fiber	Acidic pH, cGMP	Decreased	[80]
End stage-HF	Human trabeculae carneae	PKC activation	Decreased	[81]
Normal	Dog LV myofibrils	Acidic pH	Decreased	[52]
Hypoxia or Ischemia	Rat-skinned right ventricular trabeculae	Presence of inorganic phosphate	Decreased	[54]

Abbreviations: ALC-1, Atrial light chain 1; AMPK γ_2_, AMP-activated protein kinase γ_2_ subunit; cGMP, Cyclic Guanosine Monophosphate; DCM, Dilated cardiomyopathy; FHC, Familial hypertrophic cardiomyopathy; HCM, Hypertrophic cardiomyopathy; HF, Heart failure; IDC, Idiopathic dilated cardiomyopathy; LV, Left ventricle; MI, Myocardial infarction; MLC-2, Myosin light chain-2; PKA, Protein kinase A; PKC, Protein kinase C; TnC, Troponin C; TnI, Troponin I; TnT, Troponin T; VLC-1, Ventricular light chain.

## Data Availability

Not applicable.

## Abbreviations

ALC-1, Atrial Light Chain 1; BLEB, Blebbistatin; Ca^2+^, Calcium; CaMKII, Calcium/Calmodulin-dependent Protein Kinase II; cAMP, Cyclic Adenosine Monophosphate; CaV1.2, Calcium Voltage-gated Channel 1.2; cMyBP-C, Cardiac Myosin Binding Protein C; CICR, Calcium-Induced Calcium Release; DADs, Delayed Afterdepolarizations; DCM, Dilated Cardiomyopathy; EAD, Early Afterdepolarization; EC, Excitation-Contraction; EF, Ejection Fraction; ESC, European Society of Cardiology; FDA, Food and Drug Administration; HCM, Hypertrophic Cardiomyopathy; HF, Heart Failure; HFmrEF, HF with Mid-Range Ejection Fraction; HFpEF, HF with Preserved Ejection Fraction; HFrEF, HF with Reduced Ejection Fraction; IDC, Idiopathic Dilated Cardiomyopathy; IP3R2, Inositol 1,4,5-trisphosphate Receptor Type 2; Kd, Dissociation Constant; LTCCs, L-Type Ca^2+^ Channels; LV, Left Ventricle; LVOT, LV Outflow Tract; MLC-2, Myosin Light Chain-2; MYH7, β-Myosin Heavy Chain Gene; NCX, Na^+^/Ca^2+^ Exchanger; PDE3A, Phosphodiesterase 3A; PKA, Protein Kinase A; PKC, Protein Kinase C; PKD, Protein Kinase D; PKG, Protein Kinase G; PLN, Phospholamban; RV, Right Ventricle; RyR, Ryanodine Receptors; SERCA, Sarco/Endoplasmic Reticulum Ca^2+^-ATPase; SR, Sarcoplasmic Reticulum; T-tubules, Transverse Tubules; TnC, Troponin C; TnI, Troponin I; TnT, Troponin T; TRP Channels, Transient Receptor Potential Channels.

## References

[1] Virani S.S., Alonso A., Benjamin E.J., Bittencourt M.S., Callaway C.W., Carson A.P., Chamberlain A.M., Chang A.R., Cheng S., Delling F.N. (2020). Heart disease and stroke statistics—2020 update: A report from the american heart association. Circulation.

[2] Tsao C.W., Aday A.W., Almarzooq Z.I., Alonso A., Beaton A.Z., Bittencourt M.S., Boehme A.K., Buxton A.E., Carson A.P., Commodore-Mensah Y. (2022). Heart disease and stroke statistics—2022 update: A report from the American Heart Association. Circulation.

[3] Lindenfeld J., Albert N.M., Boehmer J.P., Collins S.P., Ezekowitz J.A., Givertz M.M., Katz S.D., Klapholz M., Moser D.K., Rogers J.G. (2010). HFSA 2010 comprehensive heart failure practice guideline. J. Card. Fail..

[4] Members W.C., Hunt S.A., Abraham W.T., Chin M.H., Feldman A.M., Francis G.S., Ganiats T.G., Jessup M., Konstam M.A., Mancini D.M. (2009). 2009 focused update incorporated into the ACC/AHA 2005 guidelines for the diagnosis and management of heart failure in adults: A report of the american college of cardiology foundation/american heart association task force on practice guidelines: Developed in collaboration with the international society for heart and lung transplantation. Circulation.

[5] Dharmarajan K., Rich M.W. (2017). Epidemiology, pathophysiology, and prognosis of heart failure in older adults. Heart Fail. Clin..

[6] Gazewood J.D., Turner P.L. (2017). Heart Failure with Preserved Ejection Fraction: Diagnosis and Management. Am. Fam. Physician.

[7] Pieske B., Tschöpe C., De Boer R.A., Fraser A.G., Anker S.D., Donal E., Edelmann F., Fu M., Guazzi M., Lam C.S. (2019). How to diagnose heart failure with preserved ejection fraction: The HFA–PEFF diagnostic algorithm: A consensus recommendation from the Heart Failure Association (HFA) of the European Society of Cardiology (ESC). Eur. Heart J..

[8] Satpathy C., Mishra T.K., Satpathy R., Satpathy H.K., Barone E. (2006). Diagnosis and management of diastolic dysfunction and heart failure. Am. Fam. Physician.

[9] Shah S.J., Katz D.H., Selvaraj S., Burke M.A., Yancy C.W., Gheorghiade M., Bonow R.O., Huang C.-C., Deo R.C. (2015). Phenomapping for novel classification of heart failure with preserved ejection fraction. Circulation.

[10] Ponikowski P., Voors A.A., Anker S.D., Bueno H., Cleland J.G., Coats A.J., Falk V., González-Juanatey J.R., Harjola V.-P., Jankowska E.A. (2016). 2016 ESC Guidelines for the diagnosis and treatment of acute and chronic heart failure. Kardiol. Pol..

[11] Yancy C.W., Jessup M., Bozkurt B., Butler J., Casey D.E., Drazner M.H., Fonarow G.C., Geraci S.A., Horwich T., Januzzi J.L. (2013). 2013 ACCF/AHA guideline for the management of heart failure: Executive summary: A report of the American College of Cardiology Foundation/American Heart Association Task Force on practice guidelines. Circulation.

[12] Lompré A.-M., Hajjar R.J., Harding S.E., Kranias E.G., Lohse M.J., Marks A.R. (2010). Ca^2+^ cycling and new therapeutic approaches for heart failure. Circulation.

[13] Bers D.M. (2006). Altered cardiac myocyte Ca regulation in heart failure. Physiology.

[14] Chung J.-H., Biesiadecki B.J., Ziolo M.T., Davis J.P., Janssen P.M. (2016). Myofilament calcium sensitivity: Role in regulation of in vivo cardiac contraction and relaxation. Front. Physiol..

[15] Wen Y., Pinto J.R., Gomes A.V., Xu Y., Wang Y., Wang Y., Potter J.D., Kerrick W.G.L. (2008). Functional consequences of the human cardiac troponin I hypertrophic cardiomyopathy mutation R145G in transgenic mice. J. Biol. Chem..

[16] Sommese R.F., Nag S., Sutton S., Miller S.M., Spudich J.A., Ruppel K.M. (2013). Effects of troponin T cardiomyopathy mutations on the calcium sensitivity of the regulated thin filament and the actomyosin cross-bridge kinetics of human β-cardiac myosin. PLoS ONE.

[17] Du C.-K., Morimoto S., Nishii K., Minakami R., Ohta M., Tadano N., Lu Q.-W., Wang Y.-Y., Zhan D.-Y., Mochizuki M. (2007). Knock-in mouse model of dilated cardiomyopathy caused by troponin mutation. Circ. Res..

[18] Blanchard E., Seidman C., Seidman J., LeWinter M., Maughan D. (1999). Altered crossbridge kinetics in the αMHC403/+ mouse model of familial hypertrophic cardiomyopathy. Circ. Res..

[19] Palmer B.M., Wang Y., Teekakirikul P., Hinson J.T., Fatkin D., Strouse S., VanBuren P., Seidman C.E., Seidman J.G., Maughan D.W. (2008). Myofilament mechanical performance is enhanced by R403Q myosin in mouse myocardium independent of sex. Am. J. Physiol. Heart Circ. Physiol..

[20] Chuan P., Sivaramakrishnan S., Ashley E.A., Spudich J.A. (2012). Cell-intrinsic functional effects of the α-cardiac myosin Arg-403-Gln mutation in familial hypertrophic cardiomyopathy. Biophys. J..

[21] Kim S.-J., Iizuka K., Kelly R.A., Geng Y.-J., Bishop S.P., Yang G., Kudej A., McConnell B.K., Seidman C.E., Seidman J.G. (1999). An α-cardiac myosin heavy chain gene mutation impairs contraction and relaxation function of cardiac myocytes. Am. J. Physiol. Heart Circ. Physiol..

[22] Szczesna-Cordary D., Guzman G., Zhao J., Hernandez O., Wei J., Diaz-Perez Z. (2005). The E22K mutation of myosin RLC that causes familial hypertrophic cardiomyopathy increases calcium sensitivity of force and ATPase in transgenic mice. J. Cell Sci..

[23] Szczesna-Cordary D., Jones M., Moore J.R., Watt J., Kerrick W.G.L., Xu Y., Wang Y., Wagg C., Lopaschuk G.D. (2007). Myosin regulatory light chain E22K mutation results in decreased cardiac intracellular calcium and force transients. FASEB J..

[24] Berridge M.J., Bootman M.D., Roderick H.L. (2003). Calcium signalling: Dynamics, homeostasis and remodelling. Nat. Rev. Mol. Cell Biol..

[25] Huxley A.F. (1957). Muscle structures and theories of contraction. Progr. Biophys. Chem..

[26] Hibberd M., Jewell B. (1982). Calcium-and length-dependent force production in rat ventricular muscle. J. Physiol..

[27] Kentish J.C., Ter Keurs H., Ricciardi L., Bucx J., Noble M. (1986). Comparison between the sarcomere length-force relations of intact and skinned trabeculae from rat right ventricle. Influence of calcium concentrations on these relations. Circ. Res..

[28] Patterson S.W., Piper H., Starling E. (1914). The regulation of the heart beat. J. Physiol..

[29] Solaro R.J. (2011). Modulation of cardiac myofilament activity by protein phosphorylation. Compr. Physiol..

[30] Mope L., McClellan G.B., Winegrad S. (1980). Calcium sensitivity of the contractile system and phosphorylation of troponin in hyperpermeable cardiac cells. J. Gen. Physiol..

[31] Zhang R., Zhao J., Mandveno A., Potter J.D. (1995). Cardiac troponin I phosphorylation increases the rate of cardiac muscle relaxation. Circ. Res..

[32] Li L., Desantiago J., Chu G., Kranias E.G., Bers D.M. (2000). Phosphorylation of phospholamban and troponin I in β-adrenergic-induced acceleration of cardiac relaxation. Am. J. Physiol. Heart Circ. Physiol..

[33] Haworth R.S., Cuello F., Herron T.J., Franzen G., Kentish J.C., Gautel M., Avkiran M. (2004). Protein kinase D is a novel mediator of cardiac troponin I phosphorylation and regulates myofilament function. Circ. Res..

[34] Lim C.C., Yang H., Yang M., Wang C.-K., Shi J., Berg E.A., Pimentel D.R., Gwathmey J.K., Hajjar R.J., Helmes M. (2008). A novel mutant cardiac troponin C disrupts molecular motions critical for calcium binding affinity and cardiomyocyte contractility. Biophys. J..

[35] Saad N.S., Elnakish M.T., Brundage E.A., Biesiadecki B.J., Kilic A., Ahmed A.A., Mohler P.J., Janssen P.M. (2018). Assessment of PKA and PKC inhibitors on force and kinetics of non-failing and failing human myocardium. Life Sci..

[36] Anderson P., Malouf N., Oakeley A., Pagani E., Allen P. (1991). Troponin T isoform expression in humans. A comparison among normal and failing adult heart, fetal heart, and adult and fetal skeletal muscle. Circ. Res..

[37] Solaro R.J., Powers F.M., Gao L., Gwathmey J.K. (1993). Adaptive and Maladaptive Processes: Control of Myofilament Activation in Heart Failure. Circulation.

[38] Mesnard-Rouiller L., Mercadier J.-J., Butler-Browne G., Heimburger M., Logeart D., Allen P.D., Samson F. (1997). Troponin T mRNA and protein isoforms in the human left ventricle: Pattern of expression in failing and control hearts. J. Mol. Cell. Cardiol..

[39] Van der Velden J., Klein L., Van Der Bijl M., Huybregts M., Stooker W., Witkop J., Eijsman L., Visser C., Visser F., Stienen G. (1999). Isometric tension development and its calcium sensitivity in skinned myocyte-sized preparations from different regions of the human heart. Cardiovasc. Res..

[40] Morano I., Hädicke K., Haase H., Böhm M., Erdmann E., Schaub M.C. (1997). Changes in essential myosin light chain isoform expression provide a molecular basis for isometric force regulation in the failing human heart. J. Mol. Cell. Cardiol..

[41] Miyata S., Minobe W., Bristow M.R., Leinwand L.A. (2000). Myosin heavy chain isoform expression in the failing and nonfailing human heart. Circ. Res..

[42] Margossian S.S., Anderson P.A., Chantler P.D., Deziel M., Umeda P.K., Stafford W.F., Norton P., Malhotra A., Yang F., Caulfield J.B. (1999). Calcium regulation in the human myocardium affected by dilated cardiomyopathy: A structural basis for impaired Ca^2+^-sensitivity. Mol. Cell. Biochem..

[43] Harding S.E., Brown L.A., Wynne D.G., Davies C.H., Poole-Wilson P.A. (1994). Mechanisms of β adrenoceptor desensitisation in the failing human heart. Cardiovasc. Res..

[44] Pieske B., Beyermann B., Breu V., Löffler B.M., Schlotthauer K., Maier L.S., Schmidt-Schweda S., Just H.R., Hasenfuss G. (1999). Functional effects of endothelin and regulation of endothelin receptors in isolated human nonfailing and failing myocardium. Circulation.

[45] Asano K., Dutcher D.L., Port J.D., Minobe W.A., Tremmel K.D., Roden R.L., Bohlmeyer T.J., Bush E.W., Jenkin M.J., Abraham W.T. (1997). Selective downregulation of the angiotensin II AT1-receptor subtype in failing human ventricular myocardium. Circulation.

[46] Takeishi Y., Bhagwat A., Ball N.A., Kirkpatrick D.L., Periasamy M., Walsh R.A. (1999). Effect of angiotensin-converting enzyme inhibition on protein kinase C and SR proteins in heart failure. Am. J. Physiol. Heart Circ. Physiol..

[47] Bowling N., Walsh R.A., Song G., Estridge T., Sandusky G.E., Fouts R.L., Mintze K., Pickard T., Roden R., Bristow M.R. (1999). Increased protein kinase C activity and expression of Ca2+-sensitive isoforms in the failing human heart. Circulation.

[48] Neumann J., Eschenhagen T., Jones L.R., Linck B., Schmitz W., Scholz H., Zimmermann N. (1997). Increased expression of cardiac phosphatases in patients with end-stage heart failure. J. Mol. Cell. Cardiol..

[49] Willott R.H., Gomes A.V., Chang A.N., Parvatiyar M.S., Pinto J.R., Potter J.D. (2010). Mutations in Troponin that cause HCM, DCM AND RCM: What can we learn about thin filament function?. J. Mol. Cell. Cardiol..

[50] Lang R., Gomes A.V., Zhao J., Miller T., Potter J.D., Housmans P.R. (2002). Functional analysis of a troponin I (R145G) mutation associated with familial hypertrophic cardiomyopathy. J. Biol. Chem..

[51] Elliott K., Watkins H., Redwood C.S. (2000). Altered regulatory properties of human cardiac troponin I mutants that cause hypertrophic cardiomyopathy. J. Biol. Chem..

[52] Blanchard E.M., Solaro R.J. (1984). Inhibition of the activation and troponin calcium binding of dog cardiac myofibrils by acidic pH. Circ. Res..

[53] Fabiato A., Fabiato F. (1978). Effects of pH on the myofilaments and the sarcoplasmic reticulum of skinned cells from cardiace and skeletal muscles. J. Physiol..

[54] Kentish J.C. (1986). The effects of inorganic phosphate and creatine phosphate on force production in skinned muscles from rat ventricle. J. Physiol..

[55] Gao W.D., Atar D., Liu Y., Perez N.G., Murphy A.M., Marban E. (1997). Role of troponin I proteolysis in the pathogenesis of stunned myocardium. Circ. Res..

[56] Van Eyk J.E., Powers F., Law W., Larue C., Hodges R.S., Solaro R.J. (1998). Breakdown and release of myofilament proteins during ischemia and ischemia/reperfusion in rat hearts: Identification of degradation products and effects on the pCa-force relation. Circ. Res..

[57] van Der Velden J., Klein L., Zaremba R., Boontje N., Huybregts M., Stooker W., Eijsman L., De Jong J., Visser C., Visser F. (2001). Effects of calcium, inorganic phosphate, and pH on isometric force in single skinned cardiomyocytes from donor and failing human hearts. Circulation.

[58] Wolff M.R., Buck S.H., Stoker S.W., Greaser M.L., Mentzer R.M. (1996). Myofibrillar calcium sensitivity of isometric tension is increased in human dilated cardiomyopathies: Role of altered beta-adrenergically mediated protein phosphorylation. J. Clin. Investig..

[59] Van der Velden J., Boontje N., Papp Z., Klein L., Visser F., De Jong J., Owen V., Burton P., Stienen G. (2002). Calcium sensitivity of force in human ventricular cardiomyocytes from donor and failing hearts. Basic Res. Cardiol..

[60] Margossian S., White H., Caulfield J., Norton P., Taylor S., Slayter H. (1992). Light chain 2 profile and activity of human ventricular myosin during dilated cardiomyopathy. Identification of a causal agent for impaired myocardial function. Circulation.

[61] Kobayashi T., Solaro R.J. (2005). Calcium, thin filaments, and the integrative biology of cardiac contractility. Annu. Rev. Physiol..

[62] Metzger J.M., Westfall M.V. (2004). Covalent and noncovalent modification of thin filament action the essential role of troponin in cardiac muscle regulation. Circ. Res..

[63] Bodor G.S., Oakeley A.E., Allen P.D., Crimmins D.L., Ladenson J.H., Anderson P.A. (1997). Troponin I phosphorylation in the normal and failing adult human heart. Circulation.

[64] Zakhary D.R., Moravec C.S., Stewart R.W., Bond M. (1999). Protein kinase A (PKA)-dependent troponin-I phosphorylation and PKA regulatory subunits are decreased in human dilated cardiomyopathy. Circulation.

[65] van der Velden J., Papp Z., Zaremba R., Boontje N., de Jong J.W., Owen V., Burton P., Goldmann P., Jaquet K., Stienen G. (2003). Increased Ca2+-sensitivity of the contractile apparatus in end-stage human heart failure results from altered phosphorylation of contractile proteins. Cardiovasc. Res..

[66] Van der Velden J., Merkus D., Klarenbeek B., James A., Boontje N., Dekkers D., Stienen G., Lamers J., Duncker D. (2004). Alterations in myofilament function contribute to left ventricular dysfunction in pigs early after myocardial infarction. Circ. Res..

[67] Wolff M.R., Whitesell L.F., Moss R.L. (1995). Calcium sensitivity of isometric tension is increased in canine experimental heart failure. Circ. Res..

[68] Lamberts R.R., Hamdani N., Soekhoe T.W., Boontje N.M., Zaremba R., Walker L.A., De Tombe P.P., Van Der Velden J., Stienen G.J. (2007). Frequency-dependent myofilament Ca2+ desensitization in failing rat myocardium. J. Physiol..

[69] Saad N.S., Elnakish M.T., Ahmed A.A., Janssen P.M. (2018). Protein kinase A as a promising target for heart failure drug development. Arch. Med. Res..

[70] Calderone A., Bouvier M., Li K., Juneau C., de Champlain J., Rouleau J.-L. (1991). Dysfunction of the beta-and alpha-adrenergic systems in a model of congestive heart failure. The pacing-overdrive dog. Circ. Res..

[71] Marzo K.P., Frey M.J., Wilson J.R., Liang B.T., Manning D.R., Lanoce V., Molinoff P.B. (1991). Beta-adrenergic receptor-G protein-adenylate cyclase complex in experimental canine congestive heart failure produced by rapid ventricular pacing. Circ. Res..

[72] Kiuchi K., Shannon R.P., Komamura K., Cohen D.J., Bianchi C., Homcy C.J., Vatner S.F., Vatner D.E. (1993). Myocardial beta-adrenergic receptor function during the development of pacing-induced heart failure. J. Clin. Investig..

[73] Delehanty J.M., Himura Y., Elam H., Hood W.B., Liang C.-S. (1994). Beta-adrenoceptor downregulation in pacing-induced heart failure is associated with increased interstitial NE content. Am. J. Physiol. Heart Circ. Physiol..

[74] El-Armouche A., Pohlmann L., Schlossarek S., Starbatty J., Yeh Y.-H., Nattel S., Dobrev D., Eschenhagen T., Carrier L. (2007). Decreased phosphorylation levels of cardiac myosin-binding protein-C in human and experimental heart failure. J. Mol. Cell. Cardiol..

[75] Kobayashi T., Jin L., de Tombe P.P. (2008). Cardiac thin filament regulation. Pflügers Arch..

[76] Pagani E.D., Alousi A.A., Grant A.M., Older T.M., Dziuban S.W., Allen P. (1988). Changes in myofibrillar content and Mg-ATPase activity in ventricular tissues from patients with heart failure caused by coronary artery disease, cardiomyopathy, or mitral valve insufficiency. Circ. Res..

[77] Hajjar R.J., Gwathmey J.K., Briggs G., Morgan J.P. (1988). Differential effect of DPI 201-106 on the sensitivity of the myofilaments to Ca2+ in intact and skinned trabeculae from control and myopathic human hearts. J. Clin. Investig..

[78] Blair E., Redwood C., Ashrafian H., Oliveira M., Broxholme J., Kerr B., Salmon A., Östman-Smith I., Watkins H. (2001). Mutations in the γ2 subunit of AMP-activated protein kinase cause familial hypertrophic cardiomyopathy: Evidence for the central role of energy compromise in disease pathogenesis. Hum. Mol. Genet..

[79] Parvatiyar M.S., Landstrom A.P., Figueiredo-Freitas C., Potter J.D., Ackerman M.J., Pinto J.R. (2012). A mutation in TNNC1-encoded cardiac troponin C, TNNC1-A31S, predisposes to hypertrophic cardiomyopathy and ventricular fibrillation. J. Biol. Chem..

[80] Hajjar R.J., Schwinger R.H., Schmidt U., Kim C.S., Lebeche D., Doye A.A., Gwathmey J.K. (2000). Myofilament calcium regulation in human myocardium. Circulation.

[81] Gwathmey J.K., Hajjar R.J. (1990). Effect of protein kinase C activation on sarcoplasmic reticulum function and apparent myofibrillar Ca^2+^ sensitivity in intact and skinned muscles from normal and diseased human myocardium. Circ. Res..

[82] Zipes D.P., Camm A.J., Borggrefe M., Buxton A.E., Chaitman B., Fromer M., Gregoratos G., Klein G., European Heart Rhythm Association, Heart Rhythm Society (2006). ACC/AHA/ESC 2006 guidelines for management of patients with ventricular arrhythmias and the prevention of sudden cardiac death: A report of the American College of Cardiology/American Heart Association Task Force and the European Society of Cardiology Committee for Practice Guidelines (Writing Committee to Develop Guidelines for Management of Patients With Ventricular Arrhythmias and the Prevention of Sudden Cardiac Death). J. Am. Coll. Cardiol..

[83] Ter Keurs H.E., Boyden P.A. (2007). Calcium and arrhythmogenesis. Physiol. Rev..

[84] Baudenbacher F., Schober T., Pinto J.R., Sidorov V.Y., Hilliard F., Solaro R.J., Potter J.D., Knollmann B.C. (2008). Myofilament Ca ^2+^ sensitization causes susceptibility to cardiac arrhythmia in mice. J. Clin. Investig..

[85] Robinson P., Mirza M., Knott A., Abdulrazzak H., Willott R., Marston S., Watkins H., Redwood C. (2002). Alterations in thin filament regulation induced by a human cardiac troponin T mutant that causes dilated cardiomyopathy are distinct from those induced by troponin T mutants that cause hypertrophic cardiomyopathy. J. Biol. Chem..

[86] Gomes A.V., Potter J.D. (2004). Molecular and cellular aspects of troponin cardiomyopathies. Ann. N. Y. Acad. Sci..

[87] Redwood C., Lohmann K., Bing W., Esposito G.M., Elliott K., Abdulrazzak H., Knott A., Purcell I., Marston S., Watkins H. (2000). Investigation of a truncated cardiac troponin T that causes familial hypertrophic cardiomyopathy: Ca^2+^ regulatory properties of reconstituted thin filaments depend on the ratio of mutant to wild-type protein. Circ. Res..

[88] Clark K.A., Velazquez E.J. (2020). Heart failure with preserved ejection fraction: Time for a reset. JAMA.

[89] Paulus W.J., Tschöpe C. (2013). A novel paradigm for heart failure with preserved ejection fraction: Comorbidities drive myocardial dysfunction and remodeling through coronary microvascular endothelial inflammation. J. Am. Coll. Cardiol..

[90] Hamdani N., Kooij V., van Dijk S., Merkus D., Paulus W.J., Remedios C.D., Duncker D.J., Stienen G.J., van der Velden J. (2008). Sarcomeric dysfunction in heart failure. Cardiovasc. Res..

[91] Solaro R.J., Kobayashi T. (2011). Protein phosphorylation and signal transduction in cardiac thin filaments. J. Biol. Chem..

[92] Burkart E.M., Sumandea M.P., Kobayashi T., Nili M., Martin A.F., Homsher E., Solaro R.J. (2003). Phosphorylation or glutamic acid substitution at protein kinase C sites on cardiac troponin I differentially depress myofilament tension and shortening velocity. J. Biol. Chem..

[93] Belin R.J., Sumandea M.P., Kobayashi T., Walker L.A., Rundell V.L., Urboniene D., Yuzhakova M., Ruch S.H., Geenen D.L., Solaro R.J. (2006). Left ventricular myofilament dysfunction in rat experimental hypertrophy and congestive heart failure. Am. J. Physiol. Heart Circ. Physiol..

[94] Marston S.B., de Tombe P.P. (2008). Troponin phosphorylation and myofilament Ca^2+^-sensitivity in heart failure: Increased or decreased?. J. Mol. Cell. Cardiol..

[95] Hamdani N., de Waard M., Messer A.E., Boontje N.M., Kooij V., van Dijk S., Versteilen A., Lamberts R., Merkus D., Dos Remedios C. (2008). Myofilament dysfunction in cardiac disease from mice to men. J. Muscle Res. Cell Motil..

[96] Solaro R.J., van der Velden J. (2010). Why does troponin I have so many phosphorylation sites? Fact and fancy. J. Mol. Cell. Cardiol..

[97] Dong X., Sumandea C.A., Chen Y.-C., Garcia-Cazarin M.L., Zhang J., Balke C.W., Sumandea M.P., Ge Y. (2012). Augmented phosphorylation of cardiac troponin I in hypertensive heart failure. J. Biol. Chem..

[98] Zima A.V., Bovo E., Mazurek S.R., Rochira J.A., Li W., Terentyev D. (2014). Ca handling during excitation–contraction coupling in heart failure. Pflügers Arch..

[99] Louch W.E., Stokke M.K., Sjaastad I., Christensen G., Sejersted O.M. (2012). No rest for the weary: Diastolic calcium homeostasis in the normal and failing myocardium. Physiology.

[100] Go L.O., Moschella M., Watras J., Handa K., Fyfe B., Marks A. (1995). Differential regulation of two types of intracellular calcium release channels during end-stage heart failure. J. Clin. Investig..

[101] Frisk M., Ruud M., Espe E.K., Aronsen J.M., Røe Å.T., Zhang L., Norseng P.A., Sejersted O.M., Christensen G.A., Sjaastad I. (2016). Elevated ventricular wall stress disrupts cardiomyocyte t-tubule structure and calcium homeostasis. Cardiovasc. Res..

[102] Louch W.E., Bito V., Heinzel F.R., Macianskiene R., Vanhaecke J., Flameng W., Mubagwa K., Sipido K.R. (2004). Reduced synchrony of Ca^2+^ release with loss of T-tubules—A comparison to Ca^2+^ release in human failing cardiomyocytes. Cardiovasc. Res..

[103] Song L.-S., Sobie E.A., McCulle S., Lederer W., Balke C.W., Cheng H. (2006). Orphaned ryanodine receptors in the failing heart. Proc. Natl. Acad. Sci. USA.

[104] Domeier T.L., Roberts C.J., Gibson A.K., Hanft L.M., McDonald K.S., Segal S.S. (2014). Dantrolene suppresses spontaneous Ca^2+^ release without altering excitation-contraction coupling in cardiomyocytes of aged mice. Am. J. Physiol. Heart Circ. Physiol..

[105] Cooper L.L., Li W., Lu Y., Centracchio J., Terentyeva R., Koren G., Terentyev D. (2013). Redox modification of ryanodine receptors by mitochondria-derived reactive oxygen species contributes to aberrant Ca^2+^ handling in ageing rabbit hearts. J. Physiol..

[106] Primessnig U., Schönleitner P., Höll A., Pfeiffer S., Bracic T., Rau T., Kapl M., Stojakovic T., Glasnov T., Leineweber K. (2016). Novel pathomechanisms of cardiomyocyte dysfunction in a model of heart failure with preserved ejection fraction. Eur. J. Heart Fail..

[107] Howarth F., Qureshi M., Hassan Z., Al Kury L., Isaev D., Parekh K., Yammahi S., Oz M., Adrian T., Adeghate E. (2011). Changing pattern of gene expression is associated with ventricular myocyte dysfunction and altered mechanisms of Ca^2+^ signalling in young type 2 Zucker diabetic fatty rat heart. Exp. Physiol..

[108] Dibb K., Rueckschloss U., Eisner D., Isenberg G., Trafford A. (2004). Mechanisms underlying enhanced cardiac excitation contraction coupling observed in the senescent sheep myocardium. J. Mol. Cell. Cardiol..

[109] Harzheim D., Movassagh M., Foo R.S.-Y., Ritter O., Tashfeen A., Conway S.J., Bootman M.D., Roderick H.L. (2009). Increased InsP3Rs in the junctional sarcoplasmic reticulum augment Ca^2+^ transients and arrhythmias associated with cardiac hypertrophy. Proc. Natl. Acad. Sci. USA.

[110] Shorofsky S.R., Aggarwal R., Corretti M., Baffa J.M., Strum J.M., Al-Seikhan B.A., Kobayashi Y.M., Jones L.R., Wier W.G., Balke C.W. (1999). Cellular mechanisms of altered contractility in the hypertrophied heart: Big hearts, big sparks. Circ. Res..

[111] Ljubojevic S., Radulovic S., Leitinger G., Sedej S., Sacherer M., Holzer M., Winkler C., Pritz E., Mittler T., Schmidt A. (2014). Early remodeling of perinuclear Ca^2+^ stores and nucleoplasmic Ca^2+^ signaling during the development of hypertrophy and heart failure. Circulation.

[112] Ohba T., Watanabe H., Murakami M., Takahashi Y., Iino K., Kuromitsu S., Mori Y., Ono K., Iijima T., Ito H. (2007). Upregulation of TRPC1 in the development of cardiac hypertrophy. J. Mol. Cell. Cardiol..

[113] Bush E.W., Hood D.B., Papst P.J., Chapo J.A., Minobe W., Bristow M.R., Olson E.N., McKinsey T.A. (2006). Canonical transient receptor potential channels promote cardiomyocyte hypertrophy through activation of calcineurin signaling. J. Biol. Chem..

[114] Kuwahara K., Wang Y., McAnally J., Richardson J.A., Bassel-Duby R., Hill J.A., Olson E.N. (2006). TRPC6 fulfills a calcineurin signaling circuit during pathologic cardiac remodeling. J. Clin. Investig..

[115] Triposkiadis F., Briasoulis A., Sarafidis P., Magouliotis D., Athanasiou T., Paraskevaidis I., Skoularigis J., Xanthopoulos A. (2023). The Sympathetic Nervous System in Hypertensive Heart Failure with Preserved LVEF. J. Clin. Med..

[116] Hegemann N., Primessnig U., Bode D., Wakula P., Beindorff N., Klopfleisch R., Michalick L., Grune J., Hohendanner F., Messroghli D. (2021). Right-ventricular dysfunction in HFpEF is linked to altered cardiomyocyte Ca^2+^ homeostasis and myofilament sensitivity. ESC Heart Fail..

[117] Bencivenga L., Palaia M.E., Sepe I., Gambino G., Komici K., Cannavo A., Femminella G.D., Rengo G. (2021). Why do we not assess sympathetic nervous system activity in heart failure management: Might GRK2 serve as a new biomarker?. Cells.

[118] Mayor F., Murga C. (2022). G Protein-Coupled Receptor Kinases Take Central Stage. Cells.

[119] Carrier L., Mearini G., Stathopoulou K., Cuello F. (2015). Cardiac myosin-binding protein C (MYBPC3) in cardiac pathophysiology. Gene.

[120] Moss R.L., Fitzsimons D.P., Ralphe J.C. (2015). Cardiac MyBP-C regulates the rate and force of contraction in mammalian myocardium. Circ. Res..

[121] Hamdani N., Bishu K.G., von Frieling-Salewsky M., Redfield M.M., Linke W.A. (2013). Deranged myofilament phosphorylation and function in experimental heart failure with preserved ejection fraction. Cardiovasc. Res..

[122] Røe Å.T., Aronsen J.M., Skårdal K., Hamdani N., Linke W.A., Danielsen H.E., Sejersted O.M., Sjaastad I., Louch W.E. (2017). Increased passive stiffness promotes diastolic dysfunction despite improved Ca^2+^ handling during left ventricular concentric hypertrophy. Cardiovasc. Res..

[123] D’Assante R., Arcopinto M., Rengo G., Salzano A., Walser M., Gambino G., Monti M.G., Bencivenga L., Marra A.M., Åberg D.N. (2021). Myocardial expression of somatotropic axis, adrenergic signalling, and calcium handling genes in heart failure with preserved ejection fraction and heart failure with reduced ejection fraction. ESC Heart Fail..

[124] Robinson P., Griffiths P.J., Watkins H., Redwood C.S. (2007). Dilated and hypertrophic cardiomyopathy mutations in troponin and α-tropomyosin have opposing effects on the calcium affinity of cardiac thin filaments. Circ. Res..

[125] Braunwald E., Lambrew C.T., Rockoff S.D., Ross J., Morrow A.G. (1964). Idiopathic hypertrophic subaortic stenosis: I. A description of the disease based upon an analysis of 64 patients. Circulation.

[126] van Heerebeek L., Hamdani N., Falcão-Pires I., Leite-Moreira A.F., Begieneman M.P., Bronzwaer J.G., van der Velden J., Stienen G.J., Laarman G.J., Somsen A. (2012). Low myocardial protein kinase G activity in heart failure with preserved ejection fraction. Circulation.

[127] Scotcher J., Prysyazhna O., Boguslavskyi A., Kistamas K., Hadgraft N., Martin E.D., Worthington J., Rudyk O., Rodriguez Cutillas P., Cuello F. (2016). Disulfide-activated protein kinase G Iα regulates cardiac diastolic relaxation and fine-tunes the Frank–Starling response. Nat. Commun..

[128] Hamdani N., Krysiak J., Kreusser M.M., Neef S., Dos Remedios C.G., Maier L.S., Krüger M., Backs J., Linke W.A. (2013). Crucial role for Ca^2+^/calmodulin-dependent protein kinase-II in regulating diastolic stress of normal and failing hearts via titin phosphorylation. Circ. Res..

[129] Yan C., Miller C.L., Abe J.-I. (2007). Regulation of phosphodiesterase 3 and inducible cAMP early repressor in the heart. Circ. Res..

[130] Lymperopoulos A., Rengo G., Koch W.J. (2013). Adrenergic nervous system in heart failure: Pathophysiology and therapy. Circ. Res..

[131] Solaro R.J., Rüegg J. (1982). Stimulation of Ca++ binding and ATPase activity of dog cardiac myofibrils by AR-L 115BS, a novel cardiotonic agent. Circ. Res..

[132] Shettigar V., Zhang B., Little S.C., Salhi H.E., Hansen B.J., Li N., Zhang J., Roof S.R., Ho H.-T., Brunello L. (2016). Rationally engineered Troponin C modulates in vivo cardiac function and performance in health and disease. Nat. Commun..

[133] Perrone S.V., Kaplinsky E.J. (2005). Calcium sensitizer agents: A new class of inotropic agents in the treatment of decompensated heart failure. Int. J. Cardiol..

[134] Lee J.A., Allen D.G. (1990). Calcium sensitisers. BMJ.

[135] Blinks J., Endoh M. (1986). Modification of myofibrillar responsiveness to Ca++ as an inotropic mechanism. Circulation.

[136] U.S. Food and Drug Administration (2017). Novel Drug Approvals for 2017.

[137] Brixius K., Savvidou-Zaroti P., Mehlhorn U., Bloch W., Kranias E.G., Schwinger R.H. (2002). Increased Ca^2+^-sensitivity of myofibrillar tension in heart failure and its functional implication. Basic Res. Cardiol..

[138] Dou Y., Arlock P., Arner A. (2007). Blebbistatin specifically inhibits actin-myosin interaction in mouse cardiac muscle. Am. J. Physiol. Cell. Physiol..

[139] Fedorov V.V., Lozinsky I.T., Sosunov E.A., Anyukhovsky E.P., Rosen M.R., Balke C.W., Efimov I.R. (2007). Application of blebbistatin as an excitation–contraction uncoupler for electrophysiologic study of rat and rabbit hearts. Heart Rhythm..

[140] U.S. Food and Drug Administration (2022). CAMZYOS (Mavacamten): Prescribinginformation.

[141] Green E.M., Wakimoto H., Anderson R.L., Evanchik M.J., Gorham J.M., Harrison B.C., Henze M., Kawas R., Oslob J.D., Rodriguez H.M. (2016). A small-molecule inhibitor of sarcomere contractility suppresses hypertrophic cardiomyopathy in mice. Science.

[142] Olivotto I., Oreziak A., Barriales-Villa R., Abraham T.P., Masri A., Garcia-Pavia P., Saberi S., Lakdawala N.K., Wheeler M.T., Owens A. (2020). Mavacamten for treatment of symptomatic obstructive hypertrophic cardiomyopathy (EXPLORER-HCM): A randomised, double-blind, placebo-controlled, phase 3 trial. Lancet.

[143] Awinda P.O., Watanabe M., Bishaw Y., Huckabee A.M., Agonias K.B., Kazmierczak K., Szczesna-Cordary D., Tanner B.C. (2021). Mavacamten decreases maximal force and Ca^2+^ sensitivity in the N47K-myosin regulatory light chain mouse model of hypertrophic cardiomyopathy. Am. J. Physiol. Heart Circ. Physiol..

